# Design of Wearable Textile Electrodes for the Monitorization of Patients with Heart Failure

**DOI:** 10.3390/s24113637

**Published:** 2024-06-04

**Authors:** María Jesús Sánchez, Santiago J. Fernández Scagliusi, Luis Giménez-Miranda, Pablo Pérez, Francisco Javier Medrano, Alberto Olmo Fernández

**Affiliations:** 1 Departamento de Tecnología Electrónica, E.T.S. de Ingeniería Informática, Universidad de Sevilla, Avda. Reina Mercedes s/n., 41012 Seville, Spain; sanchezgarciamj17@gmail.com (M.J.S.); sanfersca@gmail.com (S.J.F.S.); pablopg@us.es (P.P.); 2 Instituto de Microelectrónica de Sevilla, IMSE-CNM, Universidad de Sevilla, CSIC. C\Américo Vespucio 28, 41092 Seville, Spain; 3 Instituto de Biomedicina de Sevilla (IBIS-US), Hospital Universitario Virgen del Rocío (HUVR), 41013 Seville, Spain; luigimmir@gmail.com (L.G.-M.); fmedrano@us.es (F.J.M.)

**Keywords:** impedance spectroscopy, wearable sensor, textile electrode, heart failure

## Abstract

Heart failure is a severe medical condition with an important worldwide incidence that occurs when the heart is unable to efficiently pump the patient’s blood throughout the body. The monitoring of edema in the lower limbs is one of the most efficient ways to control the evolution of the condition. Impedance spectroscopy has been proposed as an efficient technique to monitor body volume in patients with heart failure. It is necessary to research new wearable devices for remote patient monitoring, which can be easily worn by patients in a continuous way. In this work, we design and implement new wearable textile electrodes for the monitoring of edema evolution in patients with heart failure. Impedance spectroscopy measurements were carried out in 5 healthy controls and 2 patients with heart failure using our wearable electrodes for 3 days. The results show the appropriateness of impedance spectroscopy and our wearable electrodes to monitor body volume evolution. Impedance spectroscopy is shown to be an efficient marker of the presence of edema in heart failure patients. Initial patient positive feedback was obtained for the use of the wearable device.

## 1. Introduction

Heart failure is a long-term medical condition that occurs when the heart is unable to efficiently pump the patient’s blood throughout the body. It has an important and rising incidence in line with the world’s aging population. More than 60 million people are diagnosed as patients with heart failure, according to [1]. In many cases, the patient is hospitalized in severe situations. These situations could be avoided with the continuous monitoring of different physiological parameters of the patient, which could lead to effective preventive actions. This would also mean a significant economic cost reduction for public healthcare systems.

The most frequently presented symptoms of heart failure, such as dyspnea, asthenia, or edema, are generally a result of one of the following main features of heart failure: volume overload [2]. This is frequently observed in the lower limbs of the patient. It is necessary for clinicians to control the patient’s volume overload in order to anticipate important clinical adverse outcomes [3,4,5]. This anticipation can lead to important clinical improvements for the patient and to an important reduction in healthcare economic costs, with the prevention of new hospitalizations.

Bioimpedance measurements have long been used for the monitoring of biological and physiological parameters [6,7,8]. In the field of heart failure, electrical impedance has also been used to monitor body volume [3,4], hemodynamics [9], or the prediction of clinical risks [10]. There are some clinical devices currently used for the monitorization of electrical impedance in patients, such as Impedimed^®^ (Carlsbad, CA, USA) [9]. However, one of the most important problems faced is that current existing procedures only measure electrical impedance in the patient during the hospitalization of the patient or during visits to the hospital, and continuous remote monitoring of the patient at home is not possible. Certified clinical equipment for impedance spectroscopy is, in all cases, large and not portable [9].

Some wearable devices have recently been proposed to monitor impedance spectroscopy and estimate volume overload in patients with heart failure [2]. In this work, (VOLUM project) the AD5941 chip from Analog Devices (Wilmington, MA, USA) was used to perform impedance spectroscopy measurements. A wearable device was used, using a printed 3D fixed structure for electrodes to be worn on the patient’s ankle. Even though results were promising, further improvements were still needed, as some patients experienced problems with the current prototypes regarding their comfort while using it, and, in some cases, it was advisable to improve the flexibility and stretchability of the device to avoid possible blood circulation complications.

Current trends with wearable devices include flexible and textile electronics as an interesting way to improve the comfort and efficiency of the devices [11,12,13,14]. Textile electronics is presented as an excellent opportunity for the development and improvement of clinical wearable devices [14], improving their usability and comfortability. This fact is of special importance with conditions affecting elderly people, such as in the case of patients with heart failure.

In our work, we study the design and implementation of new wearable textile electrode devices for their use in the remote home monitoring of patients with heart failure. Initial clinical tests have been carried out with patients and healthy volunteers to monitor electrical impedance in the lower limbs during different times of the day and evaluate the appropriateness of impedance spectroscopy to monitor body volume evolution and efficiently characterize the presence of edema in patients with heart failure.

## 2. Materials and Methods

### 2.1. Establishment of the User Needs

The initial experience of patients with heart failure in the VOLUM project was reported in [2]. In this work, a printed 3D fixed structure was used to hold the electrodes. Patients and relatives reported several problems with the original design, such as the low comfort level or fear of having possible blood circulation complications in severe cases of edema. Furthermore, the original design of the device was not easily disguised in their normal clothes. In many cases, patients expressed their preference for a possible electrode that could be integrated into their normal clothes, such as in a sock or in a compression stocking.

### 2.2. Design of the Wearable Electrodes

**Figure 1:**
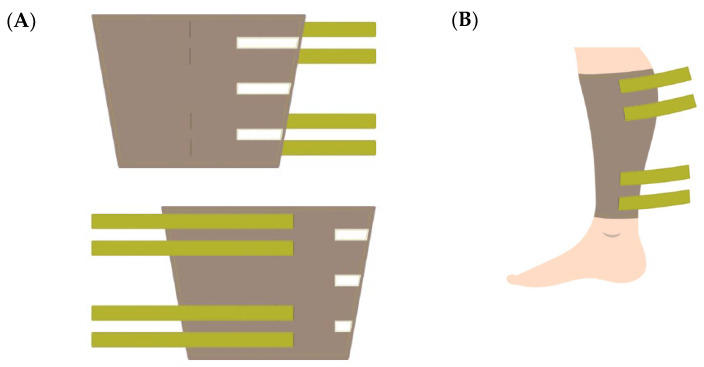
Design and implementation of the wearable device. (**A**) Design of the inner and outer surfaces of the wearable device. (**B**) The general appearance of the wearable device.

The four-electrode system, or tetrapolar configuration, was chosen for the design of the wearable electrodes. Two of the electrodes were used for current injection, and the other two electrodes for voltage measurement. This method, in opposition to bipolar measurements, did not measure electrode interface impedance together with the tissue impedance, with consequent advantages in the accuracy of the measurement, especially at low frequencies [15], while also being suitable for all frequencies. Figure 1A,B show the design of our electrode system.

### 2.3. Implementation of the Wearable Electrodes

For the fabrication of the electrode strips, Shieldex^®^ Technik-tex P130 + B (Bremen, Germany) was chosen [16]. It is a silver metalized knit composed of 22% elastomer and 78% polyamid, making it stretchable on two sides. This material was chosen for its flexibility and low electrical surface resistance (below 2 Ohms/cm according to specifications), which we thought was suitable for electrical impedance measurements.

**Figure 2:**
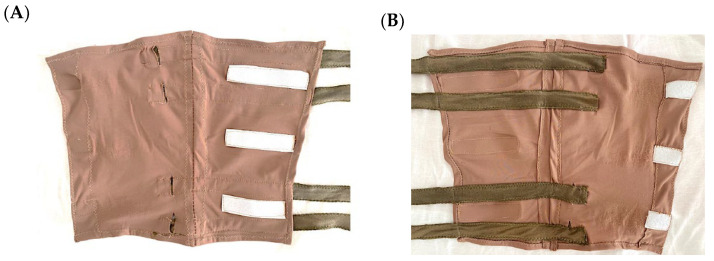
Implementation of the wearable device. (**A**) Outer surface. (**B**) Inner surface.

For the external material of the anklet, we selected an elastic fabric. Three Velcro strips along the anklet were used in order to close it and adjust it to the leg, ensuring that the strips of conductive material were well attached to the skin before making good contact with it. A user-centric design was followed. The comfort level of the patient after initial tests and previous prototypes in volunteers was taken into account to implement the final electrodes. The inner and outer surfaces of our anklet can be seen in Figure 2A,B.

### 2.4. Impedance Spectroscopy Measurement System

Wearable electrodes were designed for use with the original circuit prototype developed in the VOLUM project [17], using the AD5941 chip from Analog Devices (Wilmington, MA, USA) as the main component for bioimpedance spectroscopy measurements. This component employs the aforementioned four-wire electrode configuration setup, utilizing reduced circuitry and allowing frequency spectrometry until a frequency of 200 kHz is achieved.

For the initial tests described in our present work, the selected system for impedance spectroscopy was SFB7 from Impedimed (Carlsbad, CA, USA) [18], as it is a clinical device with the needed regulation certifications that can be used in clinical studies. It has a high degree of reliability for impedance spectroscopy measurements in patients. SFB7 scans were performed at 256 frequencies between 3 kHz and 1000 kHz. It can determine the total body of water, extracellular fluid, and intracellular fluid, using Cole modeling with the Hanai mixture theory.

### 2.5. Study Protocol

The study protocol was designed following the requirements established by local ethical committees at Hospital Universitario Virgen del Rocío of Sevilla under the VOLUM project [17].

Five healthy controls were monitored with electrodes for 3 days, at different times of the day (morning, afternoon, and night), to evaluate the dispersion of measurements and the evolution of impedance during the day. Wearable electrodes were placed 10 min before the measurements were performed, in order to ensure that the electrical response of the interfacing electrode–skin stabilized, following the recommendations of previous works, where higher impedances at the skin–electrode interface during several minutes after placement were found with textile electrodes [19,20]. Changes in condensation and skin surface conditions for an ion buffer solution were reported as a hypothesis for these variations.

Two patients were included in the study, and bioimpedance measurements were carried out on different days, in the mornings and in the afternoons. Patients were monitored during their visit to the hospital under the supervision of the research team. In a similar way to that carried out with the healthy controls, wearable electrodes were placed 10 min before the measurements to ensure impedance measurements were stabilized.

Obtained raw impedance measurements were processed to study the Cole–Cole diagram, representing the real part versus the imaginary part in impedance, and the Bode diagram, representing both the magnitude and phase of impedance versus frequency. One of the objectives of our study was to verify if the data obtained with our wearable devices were repeatable and robust enough, obtaining similar values for healthy volunteers and patients at different moments of the day. The other objective of our work was to compare the measurements between healthy individuals and patients to determine possible clinical parameters in relation to the formation of edema.

## 3. Results

**Figure 3:**
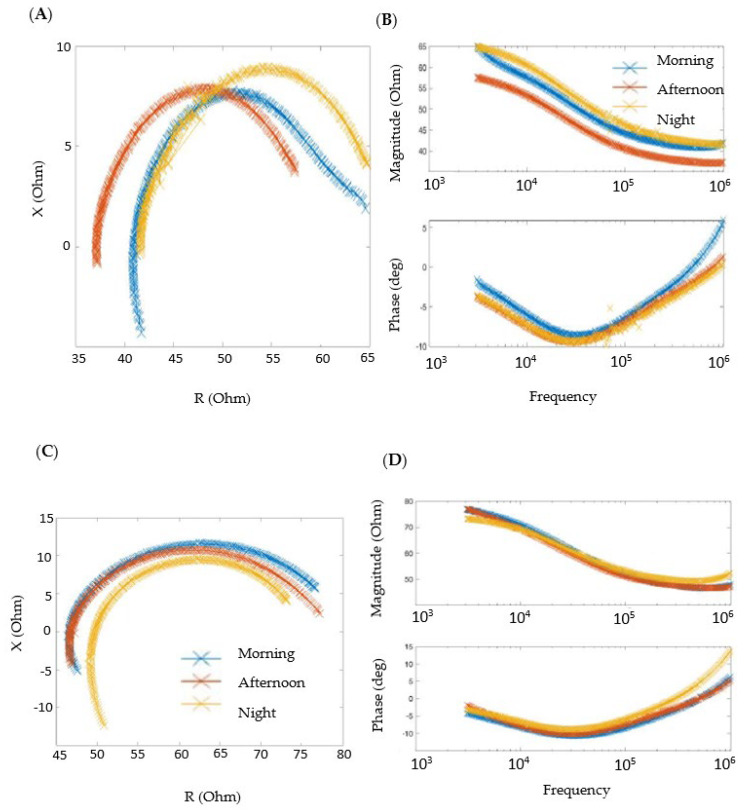
Bioimpedance data for a healthy individual at different times over one day (morning, afternoon, and night). (**A**) Cole–Cole diagram. (**B**) Bode diagram. (**C**,**D**) The bioimpedance data for the healthy individual on a different day.

Figure 3 shows the typical bioimpedance data for a healthy person at different times of the day (Cole–Cole diagram in Figure 3A and Bode diagram in Figure 3B). We observed similar values of the monitored bioimpedance during these different times (morning, afternoon, and night), showing the good repeatability and robustness of data. Another interesting result obtained was the successful comfort level of the wearable device for volunteers, even though the wearable device was not worn for a long period of time.

**Figure 4:**
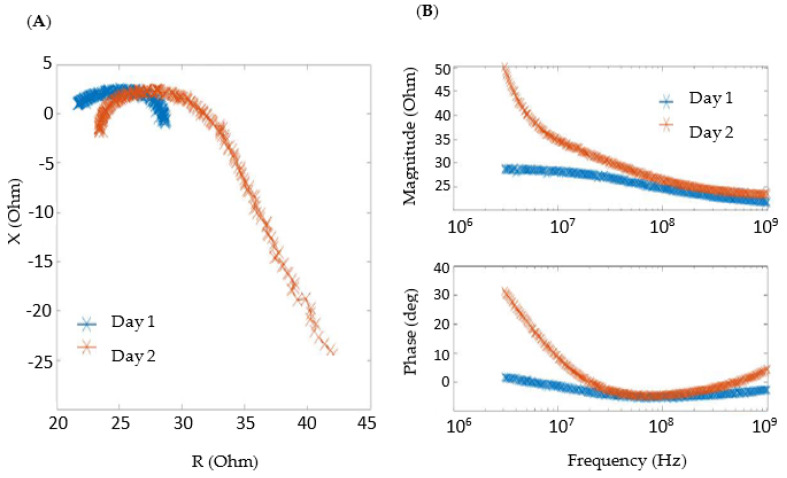
Bioimpedance typical data for patients on different days. (**A**) Cole–Cole diagram. (**B**) Bode diagram.

Figure 4 shows the typical bioimpedance data obtained for patients on different days. In a similar way to the experiments with volunteers without heart failure, impedance measurements were repeatable between days. The comfort level of the wearable electrodes was also evaluated positively by patients.

**Figure 5:**
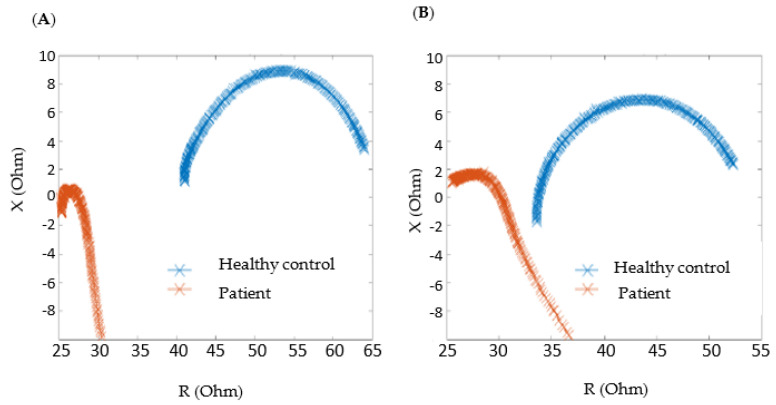
Bioimpedance data for healthy controls versus patients at different times of the day. (**A**) Morning; (**B**) afternoon.

In general, we observed lower impedance data in patients compared to healthy volunteers. This difference can be more clearly seen in Figure 5, where the typical bioimpedance data from the patients and healthy controls are shown and compared at different times of the day (morning and afternoon).

This lower impedance corresponds to the presence of edema, which was also visually seen by the physician during the clinical visit. These results are in accordance with previous reports [2,3], where a correlation between the decrease in the impedance signal and the fluid accumulation in the interstitial tissue was found due to the facility from the electric current proceeding through the extracellular medium between electrodes in the presence of edema.

## 4. Discussion

**Figure 6:**
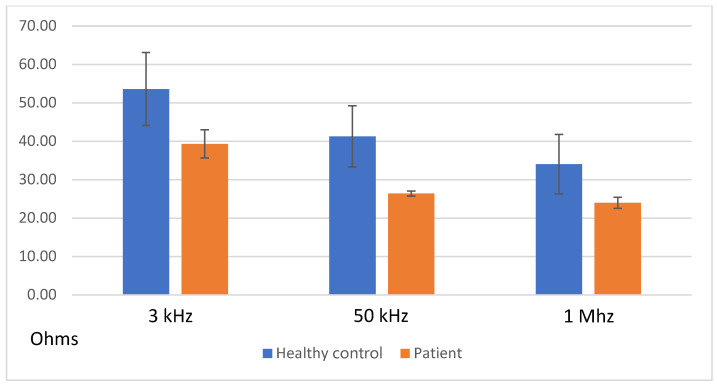
Mean value of the bioimpedance absolute value at all frequencies and dispersions.

Figure 6 illustrates the mean value of the impedance magnitude at selected frequencies as follows: 3 kHz, 50 kHz, and 1 Mhz. Data are presented for all healthy controls and patients, together with the dispersion of measurements. Together, 3 kHz and 50 kHz correspond to the lower and higher frequencies used in the SFB7 device, respectively. The value of 50 kHz corresponds to an intermediate frequency commonly used in bioimpedance measurements [2,3].

These measurements show a significant difference between the healthy controls and patients with heart failure. Lower values of the bioimpedance absolute values were found for patients with heart failure with edema. Significant differences were shown at all frequencies. The value of 50 kHz seems to be the optimal frequency for the monitoring of edema, as higher differences are seen at this frequency, and it is also a suitable frequency for the four-electrode system.

Bioimpedance measurements in the lower limbs are proven to be a useful biomarker to evaluate and potentially diagnose patients with heart failure due to their relationship with the liquid accumulation in the leg. The validity of the bioimpedance measurements is similar to the ones obtained from clinical devices [9,18], with the advantage of being wearable.

Initial feedback from patients was gathered, showing a positive opinion on the comfortability of the electrodes and the suitability to wear them for long periods of time.

### Limitations and Future Work

Future work will include the conduct of a more complete clinical study to validate the results presented in this work and obtain a robust clinical biomarker for edema that can help clinicians monitor the evolution of their patients. It will be necessary to perform the measurements of impedance spectroscopy in a continuous way, over several days, where impedance measurements can be compared to clinical evaluations during visits at the hospital, and this method can evaluate the appropriateness of impedance spectroscopy to monitor the evolution of edema in patients with heart failure.

Furthermore, it is necessary to study in a detailed and methodological way the user experience, user adoption, usability, and ergonomics. A full clinical study is being prepared under the project VOLUM, which is expected to take place in 2025.

## 5. Conclusions

In this work, we studied the use of impedance spectroscopy for monitoring the evolution of edema in patients with heart failure. We designed and implemented a novel wearable electrode system in the shape of an anklet and performed impedance spectroscopy on five healthy controls and two patients at different times of the day. The results show the robustness and repeatability of the data and the comfortability of the wearable electrodes for their use in clinical environments. Measurements show that impedance spectroscopy can be a robust potential marker for clinicians in the monitoring of the formation of edema in patients. Lower values of the measured bioimpedance are found for heart failure patients with edema. Future work will focus on conducting more complete clinical studies where our implemented electrodes can be worn in a continuous way.

## Data Availability

Data are contained within the article.
